# Vitamin D and the Development of Atopic Eczema

**DOI:** 10.3390/jcm4051036

**Published:** 2015-05-20

**Authors:** Debra J. Palmer

**Affiliations:** School of Paediatrics and Child Health, The University of Western Australia, PO Box D184, Princess Margaret Hospital, Perth WA 6001, Australia; E-Mail: debbie.palmer@uwa.edu.au; Tel.: +61-8-9340-8834

**Keywords:** eczema, infancy, pregnancy, prevention, treatment, vitamin D

## Abstract

A “vitamin D hypothesis” has been proposed to explain the increased prevalence of eczema in regions with higher latitude. This review focuses on the current available evidence with regard to the possible effect of vitamin D on the development of atopic eczema. Observational studies have indicated a link between vitamin D status and eczema outcomes, including lower serum vitamin D levels associated with increased incidence and severity of eczema symptoms. Vitamin D is known to have a regulatory influence on both the immune system and skin barrier function, both critical in the pathogenesis of eczema. However heterogeneous results have been found in studies to date investigating the effect of vitamin D status during pregnancy and infancy on the prevention of eczema outcomes. Well-designed, adequately powered, randomised controlled trials are needed. The study design of any new intervention trials should measure vitamin D levels at multiple time points during the intervention, ultraviolet (UV) radiation exposure via the use of individual UV dosimeters, and investigate the role of individual genetic polymorphisms. In conclusion, the current available evidence does not allow firm conclusions to be made on whether vitamin D status affects the development of atopic eczema.

## 1. Introduction

Changes to our modern lifestyles, with increased indoor employment and relaxation activities, along with increased sun protection behaviours, have led to limited sunlight exposure for many individuals. This can lead to vitamin D deficiency, as humans predominately derive vitamin D by cutaneous synthesis under the influence of sunlight, with limited vitamin D sourced from dietary intake [1]. The consequences of vitamin D deficiency on bone health are well established, however the influence of vitamin D status on other health outcomes has become a highly debated topic in many fields of medicine, including allergy and immunology.

A potential link between vitamin D and the development of allergic disease, the so-called “vitamin D hypothesis”, first emerged when higher rates of allergic disease were observed in higher latitudes [2,3,4,5,6] where vitamin D deficiency is more common. This latitudinal gradient effect has also been seen within countries, for example in both the USA [7] and Australia [8], there are lower rates of adrenaline auto-injector prescriptions with closer proximity to the equator. Weiland *et al.* [6] examined climatic conditions at the participating sites in the International Study of Asthma and Allergies in Childhood (ISAAC) study where patterns of global childhood eczema prevalence were determined and identified that the prevalence of eczema symptoms correlated positively with latitude and inversely with mean annual outdoor temperature. Furthermore Silverberg *et al.* [9] found the prevalence of eczema in children in the United States to be lower in regions with higher annual relative humidity, air temperature and ultraviolet (UV) index. The effect of climatic conditions on eczema symptoms were confirmed in a randomised controlled trial (RCT) by Byremo *et al.* [10], where 30 children from Norway (latitude 63°N) were sent to stay in Gran Canary (latitude 28°N) for 4 weeks in Spring or Autumn. Eczema severity significantly improved in the children who experienced the sub-tropical climate (21 to 26 °C with relative humidity 67%–72%) compared to the 26 control children who stayed at home in the subarctic/temperate climate in Norway (−5 to 21 °C with relative humidity 74%–80%). These geographical (latitude) and climatic environmental influences on eczema prevalence and severity complement the “vitamin D hypothesis” theory and this initial epidemiological evidence has since driven further observational studies and interventional trials. An increasing burden of global childhood eczema was documented by the ISAAC study, which demonstrated increased eczema prevalence in developing countries over a 10 year period to 2006 [11]. This highlights the need for effective prevention and treatment strategies. 

This review discusses the current evidence with regard to vitamin D and the development of atopic eczema, with a particular focus on the prevention of eczema in early life. MEDLINE and PUBMED database searches were performed using the keywords “atopic dermatitis” or “eczema” and “vitamin D” or “25-hydroxyvitamin D”, limiting to publication dates of 1 January 2000 to 30 November 2014, with the search also limited to human subjects and English language. Some additional original research papers were also identified through other known articles on related topics.

## 2. Pathogenesis of Eczema

Eczema is generally the first manifestation of allergic disease [12]. The natural history of this condition often follows a typical sequence, presenting as eczema in the first year of life before progressing to allergic rhinitis and asthma in subsequent years [13]. 

Observational studies have indicated a link between vitamin D status and eczema outcomes, with lower serum vitamin D concentrations associated with increased incidence, especially in children more so than in adults [14,15,16]. Interestingly variable results have been observed in relation to vitamin D status and severity of eczema symptoms, with lower vitamin D levels associated with increased severity in some studies [16,17,18], but no association with severity of symptoms in others [19,20,21]. This may potentially be explained by further characterizing the individuals with the eczema symptoms, for example both Akan *et al.* [22] and Lee *et al.* [21] observed a negative correlation between eczema severity with vitamin D levels for those study participants with allergic sensitisation but not for those without allergic sensitisation.

Recently eczema phenotypes have also been found to be associated with multiple vitamin D pathway genes [23]. Thus, vitamin D deficiency is a strong candidate in the rising predisposition to eczema. There are several biological pathways which may account for this beneficial effect of vitamin D. The pathogenesis of eczema involves dysregulation of both the immune system and skin barrier function [24], and vitamin D is known to have a regulatory influence on both of these [25]. 

Vitamin D can influence the regulation of the immune system in a number of ways. Firstly, it is believed to play an important role with regards to susceptibility to cutaneous bacterial and viral infection [24]. Individuals with eczema lack appropriate production of effectors of innate immunity, including antimicrobial peptides such as cathelicidin [26]. *In vitro* work has demonstrated that cathelicidin is induced in keratinocytes in response to vitamin D metabolites, which enhances antimicrobial activity against *Staphylococcus aureus* [26]. Moreover, vitamin D also directly suppresses skin inflammation by increasing IL-10 production by cutaneous mast cells [27]. As well as these specific cutaneous effects vitamin D also has effects on the systemic immune responses that contribute to the allergic phenotype. In particular, vitamin D receptor agonists have been shown to influence Th1 and Th2 cell function, inhibit dendritic cell maturation, induce tolerogenic dendritic cells as well as induce regulatory CD4^+^/CD25^+^/Foxp3^+^ T lymphocytes [28,29].

In terms of the skin barrier function, vitamin D and the vitamin D receptor have a regulatory role in the control of proliferation in the stratum basale, regulation of proteins in the stratum spinosum (K1, K10, involucrin) and stratum granulosum (filaggrin and loricrin), and synthesis of lipids necessary for the barrier function of the strata corneum [25]. Thus, vitamin D has the potential to modulate allergy outcomes via its multifaceted effects on altered epidermal barrier function, immune dysregulation, and inadequate bacterial defense.

## 3. Vitamin D Sources and Status

Humans naturally derive >90% of their vitamin D requirements by cutaneous synthesis under the influence of sunlight via ultraviolet B (UVB) radiation [30]. Variations in ambient UVB radiation with changes in season, latitude and air pollution can significantly impact on vitamin D levels [30], as can the magnitude of direct sun exposure on the skin influenced by skin colour, lifestyle (including time spent outdoors and sun protection practices) as well as cultural factors (customary dress). Food based dietary sources of vitamin D are limited but include deep-sea fish and cod liver oil [31]. Over the last century the use of vitamin D supplements has become more common in many populations around the world to compensate for declining natural sunlight exposure. 

If vitamin D supplementation is to be used, the ideal dose of vitamin D will depend on individual sun exposure, intake of vitamin D rich foods and current vitamin D status. The best indicator of vitamin D status is considered the measurement of serum 25-hydroxyvitamin D (25(OH)D) which is the most abundant and stable vitamin D metabolite [32]. Serum 25(OH)D concentrations reflect total vitamin D intake from sunlight exposure, dietary intake and any vitamin D supplement use. It has been well established that serum 25(OH)D levels ≥50nmol/L facilitate optimal bone health (calcitropic function of vitamin D) [33]. However in recent years there has been much discussion about the “ideal” 25(OH)D levels needed for other non-calcitropic health outcomes, including reducing the risk of allergic disease. It is important to remember that there is a potential for vitamin D toxicity if 25(OH)D is >250 nmol/L. This is usually asymptomatic but can result in hypercalcaemia, hypercalciuria and nephrocalcinosis [34]. Individual susceptibility to hypercalcaemia and vitamin D intoxication is also affected by genetic factors, such as mutations in the CYP24A1 gene [35].

Other potentially linked outcomes are eczema, vitamin D status and bone health, especially given the regular use of corticosteroids (topical and systemic), chronic inflammation and possible low vitamin D status of eczema affected individuals. A recent large adult study in the USA has observed a higher risk of fractures amongst patients with eczema [15]. Furthermore, adult eczema was associated with lower bone mineral density at the femur and spine and osteoporosis of the trochanter, but interestingly was not associated with vitamin D status [15]. Further studies investigating eczema, vitamin D status and multiple bone health outcomes appear warranted. 

## 4. Vitamin D Status *in Utero* and Eczema Development

Some observational studies [36,37] support a protective relationship between vitamin D status *in utero* and the risk of eczema development, while others suggest that high levels may be a risk factor [38]. However these studies vary with regard to the methods used to determine the possible associations between vitamin D and eczema outcomes, including estimating maternal vitamin D dietary intake in pregnancy or measuring 25(OH)D levels during pregnancy or in cord blood.

During pregnancy, fetal vitamin D levels are determined by maternal vitamin D status and the ability of 25(OH)D to cross the placenta [39]. Investigating maternal vitamin D intakes from food consumption during pregnancy, Miyake *et al.* [40] found higher dietary intakes of vitamin D to be protective against eczema symptoms in the first 2 years of life. Using this methodological approach has limitations as dietary associations may also be confounded by other immunomodulatory nutrients in whole foods. For example, one of the most important dietary sources of vitamin D intake is fatty fish, which also provide a rich source of omega-3 long-chain polyunsaturated fatty acids. These fatty acids are known to have protective effects against the development of allergen sensitisation and allergic disease [41,42,43,44]. Illustrating this point, Miyake *et al.* [40] found that the protective association between maternal vitamin D intake and eczema in the offspring was no longer significant after adjusting for associated variations in omega-3 long-chain polyunsaturated fatty acid intake. Furthermore, given that dietary sources generally contribute less than 10% of human vitamin D status, association studies based on dietary vitamin D intake without measuring individual UV radiation exposure or direct measurements of 25(OH)D levels should be interpreted with caution.

Measuring maternal 25(OH)D levels during pregnancy is a better methodological approach to determine the degree of vitamin D exposure to the developing fetus *in utero*. Table 1 summarizes the observational studies which have measured maternal 25(OH)D levels during pregnancy [38,45,46] and/or in cord blood [36,37,45,47] at birth and reported on eczema outcomes in the off-spring. A major limitation of these studies is that 25(OH)D levels were only measured once, thus failing to capture the effects of likely changes in 25(OH)D status and exposure to the fetus over the course of pregnancy. Future studies should be designed to assess dynamic changes in vitamin D status, by measuring 25(OH)D levels at least once in each trimester of pregnancy (every 3 months) as well as cord blood, to capture seasonal variations and changes to diet or use of vitamin D supplementation practices during the *in utero* period. The heterogeneous findings with regard to maternal antenatal or cord blood 25(OH)D status and off spring eczema outcomes from observational studies illustrates the need for well-designed RCTs on this topic.

To date there has only been one published intervention trial investigating the effect of vitamin D exposure *in utero* on the risk of allergic disease development in the offspring.

This small (*n* = 180) RCT conducted by Goldring *et al.* [48] in the United Kingdom (latitude 51°N) investigated the effect of two different vitamin D supplements during the third trimester of pregnancy (from 27 weeks gestation) and found no differences in eczema outcomes in the children by three years of age. Goldring *et al.* [48] compared a control group (no vitamin D supplementation) with two intervention groups receiving 800 IU/day ergocalciferol or a single bolus of 200,000 IU cholecalciferol. In humans, cholecalciferaol contributes 80% to 90% of the total 25(OH)D concentration and is thought to be more potent than ergocalciferol [49]. Interestingly, the median (inter quartile) cord blood 25(OH)D levels of both intervention groups in the Goldring study [48] were similar at 26 nmol/L (17–45 nmol/L) for the ergocalciferol group compared to 25 nmol/L (18–34 nmol/L) for the cholecalciferol group. These cord blood 25(OH)D levels were similar to those of a German cohort study (27 nmol/L, 17–43 nmol/L) [45] conducted at the same latitude of 51°N. This study also found no association with offspring eczema outcomes. However, both of these average cord blood 25(OH)D levels were low in comparison to the lower latitude cohort studies [36,37] which found an association of higher cord blood 25(OH)D levels with reduced risk of child eczema outcomes (see Table 1). Hence given the higher latitude and resulting lower ambient UV radiation exposure of the Goldring study setting [48], the results from this RCT should be interpreted with caution as the supplementation doses (800 IU/day ergocalciferol or a single bolus of 200,000 IU cholecalciferol) of the intervention groups may have been insufficient for an eczema prevention strategy in this study population. 

Currently there are several other RCTs also investigating the effect of maternal vitamin D supplementation during pregnancy on allergic disease outcomes (NCT00920621 and NCT00856947). These RCTs are larger (*n* = 600–870 women) and are using higher doses (2400 IU or 4000 IU per day) of maternal vitamin D supplementation during pregnancy. Over the next few years the results of these trials should clarify any effect of vitamin D during pregnancy on early childhood allergic disease.

**Table 1:** Observational studies investigating maternal antenatal and/or cord blood 25(OH)D levels and eczema outcomes.

Reference and Study Location	Study Design	Study Population and Latitude	25(OH)D Levels	Main Results	Higher Vitamin D Level
Gale *et al.* 2008 [38] Southampton, UK	Prospective birth cohort	466 mother-child pairs Latitude 51°N	Maternal antenatal blood during third trimester of pregnancy median = 50 nmol/L (IQR 30–75)	Higher maternal 25(OH)D levels of >75 nmol/L (compared with lower levels <30 nmol/L) were associated with an increased risk of eczema at 9 months of age	Eczema ^c^
Weisse *et al.* 2013 [45] Leipzig, Germany	Prospective birth cohort	378 mother-child pairs Latitude 51°N	Maternal antenatal blood during third trimester of pregnancy median = 55 nmol/L (IQR 36–78)	No associations between maternal antenatal 25(OH)D levels with risk of eczema in the children to 2 years of age.	Eczema ^b^
Wills *et al.* 2013 [46] South West of England	Prospective birth cohort	5513 mother-child pairs Latitude 51°N	Maternal antenatal blood during first, second or third trimester of pregnancy median = 62 nmol/L (IQR 46–81)	No associations between maternal antenatal 25(OH)D levels with risk of eczema in the children at 7 years of age.	Eczema ^b^
Baiz *et al.* 2014 [36] Poitiers and Nancy, France	Prospective birth cohort	239 mother-child pairs. Latitude 46–48°N	Cord blood mean = 44 nmol/L (IQR = 38 nmol/L)	Inverse association between cord blood 25(OH)D levels with eczema in the children by age 1, 3, 5 years.	Eczema ^a^
Chawes *et al.* 2014 [47] Copenhagen, Denmark	Prospective birth cohort	257 mother-child pairs Latitude 55°N	Cord blood median = 48 nmol/L	No association between cord blood 25(OH)D levels with eczema in the children to 6–7 years old.	Eczema ^b^
Jones *et al.* 2012 [37] Perth, Australia	Prospective birth cohort	231 mother-child pairs Latitude 32°S	Cord blood mean (SD) = 58 nmol/L ± 24.1 nmol/L	Inverse association between cord blood 25(OH)D levels with risk for eczema in the children at 12 months of age.	Eczema ^a^
Weisse *et al.* 2013 [45] Leipzig, Germany	Prospective birth cohort	378 mother-child pairs Latitude 51°N	Cord blood median = 27 nmol/L (IQR 17–43)	No associations between cord blood 25(OH)D levels with risk of eczema in the children to 2 years of age.	Eczema ^b^

^a^ Inverse association; ^b^ no association; ^c^ positive association.

## 5. Vitamin D Status in Early Infancy and Eczema Development

Breast milk, despite its many other benefits, has variable but generally low 25(OH)D levels of around 25 IU/L from lactating women who have “sufficient” maternal 25(OH)D status [50]. However breast milk 25(OH)D levels can be improved via increased maternal sunlight exposure [51] and/or maternal oral vitamin D supplementation during lactation [52]. For infants who have minimal sun exposure themselves, it has been estimated that in order to achieve an infant intake of 400–500 IU per day, the lactating mother would need to ingest or acquire through UV exposure 6000 IU per day lactation [52]. This is consistent with two studies which have found maternal doses of 4000–6000 IU per day of oral vitamin D during lactation may be needed to achieve infant 25(OH)D levels ≥50 nmol/L [53,54]. Breastfed infants who have limited sun exposure can also be given oral vitamin D supplementation directly. Recent randomised trials [55,56] comparing different doses, 200 IU, 400 IU, 800 IU, 1200 IU or 1600 IU per day of infant oral vitamin D supplementation determined that a dose of 400 IU per day resulted in >95% infants achieving vitamin D sufficiency with a 25(OH)D level of ≥50 nmol/L and has a reduced risk of hypercalcaemia compared to higher doses. This dose is consistent with current infant vitamin D supplementation guidelines from Europe [57], Canada [58] and the United States [31]. 

From the 1920s to the present, the use of vitamin D supplementation has been routinely recommended for the prevention of rickets in many countries particularly in the Northern Hemisphere [31,57,58]. However, despite routine vitamin D supplementation in many countries, there are limited studies investigating the effect of oral vitamin D supplementation and allergic disease outcomes in childhood. One Swedish prospective birth cohort study [59] found that higher intake of dietary vitamin D (>524 IU/day) during the first year of life was correlated with an increased risk of eczema to 6 years of age. This study [59] estimated infant vitamin D intake using dietary questionnaires at 5, 7 and 10 months of age, and included vitamin D intakes from breast milk, infant formula and solid foods, as well as use of vitamin D supplementation. However no 25(OH)D levels were measured and the sample size was small (*n* = 123 infants) placing major limitations on the interpretation of these findings. 

In order to definitively determine whether postnatal vitamin D supplementation has an effect on childhood eczema development well-designed adequately powered randomised controlled trials are essential. The study design of any new intervention trial in this field should measure 25(OH)D levels at multiple time points during the intervention and follow-up period, and ideally also measure UVB radiation exposure via the use of individual UV dosimeters. 

## 6. Vitamin D as a Treatment Approach for Eczema Symptoms

UV radiation phototherapy as a treatment for moderate to severe eczema symptoms in adults has been appreciated for many years [60]. However due to concerns regarding the potential for increased skin cancer risk when using a UV radiation exposure approach, along with increased recognition of lower vitamin D levels associated with increased severity of eczema symptoms [16,17,18], several recent RCTs have investigated oral supplementation with vitamin D as an alternative eczema treatment.

Maternal vitamin D supplementation in the postnatal period was studied in a RCT [61] conducted in Japan. This trial assessed the effects of maternal vitamin D supplementation with 800 IU vitamin D per day (*n* = 82) or placebo (*n* = 82) for 6 weeks in lactating mothers of infants who had facial eczema by one month of age. There were no differences between the groups in eczema severity at 3 months or incidence of eczema at the two year of age follow-up assessment. Unfortunately, any interpretation of the findings from this trial are particularly limited as serum 25(OH)D levels were not measured in either the mothers or the infants. This was a critical study design flaw given that two studies [53,54] have found that maternal vitamin D supplementation during lactation requires higher doses of 4000–6000 IU per day to achieve infant 25(OH)D levels ≥50 nmol/L. Another major criticism of this RCT [61] was that some of the infants were fully breastfed while others were only partially breastfed and consumed infant formula, which can also be a major dietary source of vitamin D supplementation (360–520 IU/L). Potential differences in the volumes of formula consumed were not described. The use of maternal vitamin D supplementation during lactation still requires further investigation with well-designed intervention trials before this can be recommended or disregarded as a treatment strategy for eczema in breastfed infants.

Promising results of improved eczema symptoms have been found using oral vitamin D supplementation (1000 IU vitamin D for 1 month) in children, aged 2–17 years, during winter in two double-blinded placebo-controlled RCTs [62,63]. Both of these trials were conducted at northern latitudes of 42°N (Boston, MA, USA) and 48°N (Ulaanbaatar, Mongolia), where lower vitamin D status in winter was assumed. However, serum 25(OH)D levels were not measured in the children in either of these trials. It is also important to note that the participants in both of these trials were selected for a history of winter-time exacerbation of eczema, hence this potential treatment strategy may not be as effective on all children with eczema.

In adults, mixed results have been found in several randomised, placebo-controlled, double-blind trials investigating vitamin D supplementation as a treatment strategy for atopic dermatitis. Using an oral vitamin D supplement of 1600 IU per day for 60 days, both Amestejani *et al.* [64] and Javanbakht *et al.* [65] found significant improvement in atopic dermatitis severity scores in the vitamin D treatment group compared to the placebo group, whereas Hata *et al.* [66] determined that using a higher dose of 4000 IU per day but for only 21 days did not improve eczema extent and severity scores in their study population. The use of oral vitamin D supplementation as an adjunctive treatment for atopic eczema/dermatitis in both children and adults warrants further trials to identify which dose and duration of treatment is ideal especially among different racial populations living at various latitudes and through different seasons.

Calcipotriene is a topical vitamin D3 analogue approved for the treatment of scalp psoriasis. After a case of eczema flare in response to application of calcipotriene cream in a 2 year old boy, Turner *et al.* [67] investigated calcipotriene application in a mouse model of atopic dermatitis. They found that the mice developed a persistent eczematous dermatitis at sites of calcipotriene application. This was consistent with earlier work by Li *et al.* [68,69]. Hence at present the use of topical vitamin D3 analogues for the treatment of eczema symptoms cannot be recommended.

## 7. Vitamin D Status and Allergen Sensitisation

With regard to the possible effects of vitamin D on allergen sensitisation, again we find heterogeneous results from observational studies. Some studies have found higher maternal vitamin D intake from foods during pregnancy [70] or higher infant vitamin D levels were associated with reduced allergen sensitisation in childhood [17], whereas Weisse *et al.* [45] found that higher maternal 25(OH)D levels, with a median of 55 nmol/L during third trimester of pregnancy, were associated with increased risk of food allergen sensitization by 2 years of age. No associations between cord blood 25(OH)D levels and off-spring allergen sensitisation have been shown in numerous cohort studies [37,45,47] or the RCT of Goldring *et al.* [48]. 

Some of these “inconsistent” results may be explained by possible “U-shaped associations”, with an increased risk of allergen sensitisation at both low and high levels of vitamin D. This is well illustrated in a study by Rothers *et al.* [71] which observed an increased risk of aeroallergen sensitization in children who had both low (<50 nmol/L) and high (≥100 nmol/L) levels of cord blood 25(OH)D. A similar U-shaped effect has been observed in other epidemiological studies where the risk of elevated IgE concentrations is increased for both low and high 25(OH)D levels [72]. A potential explanation for this phenomenon is that 25(OH)D may promote a TH2 immune skew at the systemic level, while simultaneously promoting an anti-inflammatory effect at local organ level. The balance between these effects may vary at different 25(OH)D levels [73].

## 8. Future Directions

### 8.1. Genotype Variations

In depth genotyping with regard to eczema, IgE synthesis and vitamin D metabolism will likely to be able to shed more light and provide further advances in our understanding of the role of vitamin D on the development of atopic eczema. Liu *et al.* [74] identified that specific genotypes (IL4, MS4A2, FCER1G and CYP24A1) known to be involved in IgE synthesis and vitamin D metabolism, were able to modify the effects of vitamin D deficiency on the risk of food sensitisation. A subsequent study showed that the combination of low vitamin D status at birth and in early childhood in children carrying the C allele of rs2243250 was associated with a greater than threefold increased risk of food sensitisation [75].

Recently eczema phenotypes have also been found to be associated with multiple vitamin D pathway genes [23]. In a large cohort study of 1442 Chinese children with eczema and 1231 non-allergic controls, atopic eczema was associated with a vitamin D-related SNP rs4674343 on CYP27A1 (odds ratio 0.66, 95% confidence interval 0.53–0.83, *p* = 0.0004). Furthermore two CYP2R1 haplotypes increased eczema risk whereas one vitamin D receptor haplotype lowered eczema risk. GC rs7041 and CYP2R1 rs7935792 also interacted to modulate total IgE among these Chinese eczema patients. 

Heine *et al.* [76] studied the frequency of four common vitamin D receptor gene polymorphisms and found that a specific vitamin D receptor haplotype is more frequent in patients with severe atopic dermatitis. They identified that *Bsm*I (rs1544410) G allele, *Apa*I (rs7975232) C allele and *Taq*I (rs731236) T allele were all over-represented in patients with severe atopic dermatitis compared to healthy controls. Together these findings suggest that the effect of vitamin D status on allergy outcomes may be influenced by the underlying genetic milieu.

### 8.2. Vitamin D or Sunlight?

Along with genetic variability, another explanation for the heterogeneous findings with regard to the effect of vitamin D status and atopic eczema may be the source of the vitamin D. Could there be an effect of sunlight exposure or UV radiation on the skin that is independent of the parallel effect that UV radiation has on vitamin D status? An interesting finding from a recent study by Noh *et al.* [19] was that lower vitamin D levels in atopic dermatitis patients were correlated to total body area affected by eczema, but specifically the more sun exposed areas of head and neck compared to trunk. The associations at the foundation of the “vitamin D hypothesis” for allergy prevention are based on population measures of sunlight exposure, such as latitude and seasonality, so a better understanding of possible “non-vitamin D” sunlight-related immunomodulatory effects also appears to be warranted. In particular, this raises the question of other bioactive molecules, such as nitric oxide [77], which is released as a result of sunlight exposure and could be mediating effects on immune function and allergic disease development risk. There is already preliminary evidence in animal models that UVB radiation has vitamin D-independent effects on immune modulation [78], hence this line of investigation needs to be more fully explored in a clinical human context in future studies.

## 9. Conclusions

A lack of well-designed randomised controlled trials investigating the effect of vitamin D (either from foods, supplementation and/or sunlight exposure) on the development of eczema has resulted in limited and conflicting evidence to date on this topic. The sunlight exposure effect alone on the incidence and severity of eczema especially requires in detailed further examinations at various time points in early life. The use of devices such as individual UV dosimeters, which are now readily available to directly measure UVB radiation exposure, should be an essential measurement outcome in all future studies. Unlocking the role of individual genetic polymorphisms and their influence on vitamin D status in early life and eczema development will also be essential, and may be of some value in explaining the lack of consistency in findings to date. There remains enormous scope for addressing the role of these diet and lifestyle factors as potential allergy prevention strategies. To date, the current available evidence does not allow firm conclusions to be made on whether vitamin D status affects the development of atopic eczema. 
